# Application of Genomics Tools to Animal Breeding

**DOI:** 10.2174/138920212800543057

**Published:** 2012-05

**Authors:** Jack C.M. Dekkers

**Affiliations:** Department of Animal Science, Iowa State University, Ames, IA 50011, USA

**Keywords:** Animal breeding, quantitative genetics, whole genome association studies, genomic selection.

## Abstract

The main goal in animal breeding is to select individuals that have high breeding values for traits of interest as parents to produce the next generation and to do so as quickly as possible. To date, most programs rely on statistical analysis of large data bases with phenotypes on breeding populations by linear mixed model methodology to estimate breeding values on selection candidates. However, there is a long history of research on the use of genetic markers to identify quantitative trait loci and their use in marker-assisted selection but with limited implementation in practical breeding programs. The advent of high-density SNP genotyping, combined with novel statistical methods for the use of this data to estimate breeding values, has resulted in the recent extensive application of genomic or whole-genome selection in dairy cattle and research to implement genomic selection in other livestock species is underway. The high-density SNP data also provides opportunities to detect QTL and to encover the genetic architecture of quantitative traits, in terms of the distribution of the size of genetic effects that contribute to trait differences in a population. Results show that this genetic architecture differs between traits but that for most traits, over 50% of the genetic variation resides in genomic regions with small effects that are of the order of magnitude that is expected under a highly polygenic model of inheritance.

## INTRODUCTION

Genetic improvement in domesticated animal populations that are used for agricultural production mainly involves selection of males and females that, when mated, are expected to produce progeny that perform better than the average of the current generation. Performance usually includes a combination of multiple characteristics, or traits, most of which are quantitative in nature. Quantitative traits are controlled by multiple to many genes (>100 to perhaps thousands), along with environment [1] and, in the case of livestock include traits such as milk yield, fat yield, protein yield and longevity in dairy cattle, and growth rate, fatness and feed intake in beef cattle and pigs. The main criteria that are used to identify individuals to be used for breeding are estimates of their breeding values for the traits of interest. The breeding value of an individual is defined as the sum of the additive effects of all loci that contribute to the trait (quantitative trait loci or QTL), deviated from the population mean [1]. Under some assumptions, this is equivalent to two times the expected phenotype of progeny deviated from the population mean [1], which is what animal breeders aim to improve. The factor two stems from the fact that progeny receive half of their alleles from their father and half from their mother. To date, extensive data bases of recorded phenotypes for traits of interest, or for traits that are genetically correlated to traits of interest, have been used as the main source of information to estimate the breeding value of selection candidates. To this end, sophisticated statistical methods based on best linear unbiased prediction (BLUP) mixed linear model methodology [2,3] have been implemented. These methods capitalize on information contained in the recorded phenotypes of not only the individual itself but also that of its relatives, in order to maximize the accuracy of the resulting estimated breeding value (EBV). Here, accuracy is defined as the correlation between the true and estimated breeding value and is one of the main determinants of the rate of genetic improvement that can be achieved in a breeding program per unit of time. Other determinants are the selection intensity, which is related to the proportion of individuals that are selected to be parents), and the age at which breeders are selected and reproduce, which defines the generation interval. The expected rate of improvement per unit of time is proportional to accuracy and selection intensity and inversely proportional to the generation interval [1].

Statistical models and selection theory used in animal breeding are based on the so-called infinitesimal genetic model of quantitative genetics [1]. The infinitesimal model assumes the trait is affected by a large (infinite) number of unlinked genes with very small and additive effects. Simulation studies have, however, shown that, at least in the short term, results from these models are rather robust to the true genetic architecture of traits [4]. As a result, specific knowledge of the genetic architecture is not essential for these phenotype-based methods to be effective.

Although selection programs based on EBV estimated from phenotype have been very successful, they also face a number of limitations. These primarily relate to the ability to routinely record phenotypes on selection candidates and/or their close relatives in a timely manner, such that accurate selection decisions can be made at an early age, the latter to reduce generation intervals. Cost of phenotype recording also plays an important role here. Unfortunately, some traits of interest are only recorded late in life (e.g. longevity), only on one sex (e.g. milk yield in dairy cattle), require animals to be sacrificed (e.g. meat quality), or require animals to be exposed to conditions that would hamper the ability to market or export their germplasm (e.g. disease resistance). These phenotyping constraints limit the amount of genetic progress that can be made. In order to overcome these limitations, animal breeders have a long history of investigating opportunities to get early measurements on selection candidates that can be used to increase the accuracy of EBV at a young age. Initial work focused on indicator traits, physiological measurements and blood markers. One of the early successes and applications was the use of blood groups as a genetic marker to select for disease resistance in chickens [5]. An example of a physiological measure is serum IGF-1 measured at an early age in cattle and pigs as an indicator of efficient growth [6]. In general, however, the use of such indicator traits, and especially physiological indicator traits measured in blood, has been limited.

Against this background, the purpose here is to review the impact that molecular genetics has had and is having on genetic improvement programs in livestock, in particular the recent availability of high-density SNP genotyping panels. In addition, the knowledge that this is providing on the genetic architecture of traits of interest will be addressed, along with implications and opportunities for the future.

## THE FIRST ERA OF MOLECULAR GENETICS

Starting in the 1970’s, the advent of the era of molecular genetics provided new opportunities to enhance breeding programs in livestock by allowing the use of DNA markers to identify genes or genomic regions that control traits of interest. The obvious first application of these methods was to discover the genetic basis and develop genetic tests for single gene defects. For quantitative traits, these advances promised the identification of QTL and the development of DNA tests that could be done on all selection candidates at an early age to help inform selection decision through marker-assisted selection (MAS), i.e. selection on a combination of information derived from genetic markers associated with QTL and the traditional phenotypic information [7,8,9]. To this end, large numbers of candidate gene and QTL mapping studies were conducted [10,11]. This resulted in the discovery of substantial numbers of QTL and marker-phenotype associations and some causative mutations [12]. The implementation of this information in breeding programs, was however limited for various reasons [12], but primarily because, 1) most QTL studies were conducted in experimental crosses to create extensive linkage disequilibrium, rather than in the populations that are used for genetic improvement, 2) most effects discovered tended to explain only a limited amount of genetic variation for the trait, 3) many QTL and associations could not be replicated, and 4) the still high cost of routine genotyping of selection candidates for even a handful of genetic markers. There are, however, some notable exceptions of the discovery of genes or markers with large effects that were repeatable and that were implemented in practical breeding programs [12].

## THE PRESENT ERA OF MOLECULAR GENETICS

### Use of High-Density SNP Genotyping for Whole-Genome Selection

For most livestock species, commercial platforms are currently available that allow the genotyping of an individual for tens of thousands of SNP across the genome at a reasonable cost (<$150 per individual, depending on volume). The first such high-density SNP genotyping platform available in livestock was the 50k Bovine Illumina SNP panel [13]. To date, tens of thousands of dairy and beef bulls and cows have been genotyped using this platform. Similar SNP panels of 40 to 65 thousand SNP are now available for other livestock species, including pigs, poultry, sheep, and horse. Recently, panels with over 700k SNP have become available in cattle and such higher density panels are also under development in other species. 

In dairy cattle, the main use of high-density SNP genotyping has been to implement genomic or whole-genome selection [14,15,16,17]. Genomic selection involves estimation of the effect of each SNP on the high-density panel using models that fit all SNP simultaneously, with their effects treated as random variables. Several Bayesian approaches have been developed to implement this statistical estimation using Monte Carlo Markov Chain (MCMC) methodology [18,19]. Methods differ in the prior assumptions that are made about the distribution of SNP effects and include Bayesian variable selection methods, in which only a proportion of the SNP is fitted in each iteration of the MCMC chain, with the remaining SNP assumed to have zero effects. This proportion of SNP with non-zero effects can either be set as a prior or estimated from the data [19]. Once estimates of the effect of each SNP are obtained, they can be used to estimate the breeding value of selection candidates based on their SNP genotypes across the genome. 

Alternatively, the high-density SNP genotypes can be used to construct a so-called genomic relationship matrix among all individuals in the population and use it instead of the traditional pedigree-based relationship matrix in the BLUP mixed model procedures that are routinely used to estimate breeding values in livestock [2]. This procedure, known as GBLUP, has been shown to be equivalent to the Bayesian SNP effect estimation method in which the prior distribution of SNP effects assumes that the genetic variation for the trait is equally distributed across all SNP on the panel, similar to the infinitesimal model of quantitative genetics [20]. Thus, in contrast to the phenotype-based models for prediction of breeding values, methods that utilize genomic data do depend on having some knowledge of the genetic architecture of traits [21,22]. Alternatively, non- or semi-parametric methods have been advocated for use in genomic selection [23,24]. Methods to combine data on genotyped individuals with phenotypic data on individuals that have not been genotyped have been developed also [25].

Estimation of breeding values using high-density SNP data has been implemented in dairy cattle breeding programs in several countries and research to implement genomic selection in other livestock species is underway [17]. In dairy cattle, this has resulted a substantial increase in the accuracy of EBV at a young age (increases of 35% on average and up to 50%, depending on the trait and size of the data set used for training) [26]. The availability of genome-enhanced breeding values (GEBV) at a young age is having a major impact on breeding programs in dairy cattle, in particular by allowing young bulls to be selected for breeding prior to the availability of extensive progeny data. This is expected to substantially increase (up to double) the rate of genetic improvement by reducing generation intervals [27] and by enhancing opportunities to select for traits with low heritability, e.g. fertility. Reduction or removal of the need for progeny testing, also has the potential to substantially reduce the cost of breeding programs in dairy cattle [28].

Implementation of genomic selection in other livestock species is still under development. In contrast to dairy cattle, breeding programs in other species offer fewer opportunities to increase rates of genetic improvement by reducing generation intervals, as selection is typically already at an early age. In addition, the large training data sets of genotyped individuals with accurate phenotypic data (e.g. progeny-test information) that are needed to obtain GEBV with sufficient accuracy are more difficult to attain in these species. Equations have been developed to predict the accuracy of GEBV as a function of the number of genotyped individuals, the accuracy of the phenotypic information available on them, and the genetic architecture of the trait [29,30,31]. These equations predict a requirement for training data sets of at least several thousand genotyped individuals with accurate phenotypes to obtain reasonable accuracy of GEBV, with greater numbers needed for traits that have a more polygenic genetic architecture and if genotyped individuals have less accurate phenotypic data available on them.

### Use of High-Density SNP Genotyping for Genome-Wide Association Studies

The large amounts of high-density SNP data that are being generated for implementation of whole-genome selection can also be used for genome-wide association studies (GWAS) to identify genetic markers or genomic regions associated with traits based on population-wide linkage disequilibrium (LD). Several studies have capitalized on this to analyze the genetic architecture of traits of interest in animal agriculture [32]. For GWAS, several alternate statistical methods have been used. Most studies have used single SNP models in which each SNP is fitted separately as a fixed effect, ideally in a BLUP animal model to properly account for the family structure of the data by fitting a polygenic effect with pedigree-based relationships [33,34,35]. A problem of single-SNP models is that they rely on the pair-wise LD of a QTL with individual SNP. Single SNP models can also lead to excessive false positives if population structure is not properly accounted for [36]. Hayes *et al.* [36] used mixed linear model methodology to estimate the proportion of genetic variance associated with each genomic region of 50 SNP from the Bovine 50k Illumina SNP chip for three quantitative traits in dairy cattle. Fitting each region separately, their model simultaneously used two genomic relationship matrices, one based on 50 SNP in the region and one based on the rest of the genome, to separate genetic variance contributed by the region from variance contributed by the rest of the genome.

The Bayesian methods that have been developed for genomic selection have also been used for GWAS. In particular the Bayesian variable selection methods have been shown to be effective for GWAS in simulated [38,39] and real data [40,41]. Several criteria have been used to identify important SNP or genomic regions using these methods, including the proportion of iterations of the MCMC chain that a given SNP or a set of SNP in a genomic region were given non-zero effects, or the proportion of variance that is explained by a given SNP or by a region of the genome [39,40,41]. An advantage of the genomic selection methods over the single SNP models is that all SNP are fitted simultaneously. This allows capture of all information if multiple SNP are in LD with a QTL and also implicitly accounts for any population structure that is present in the data, reducing false positives. In addition, by fitting SNP effects as random rather than fixed, estimates are shrunk towards zero depending on the amount of information that is contained in the data and the priors that are specified. A yet unresolved question is the impact of the choice of priors. Also, satisfactory criteria to identify important SNP or genomic regions have not been fully developed for these methods. For example, Fan *et al*. [40] and Onteru *et al*. [41] used bootstrapping of genomic regions of interest to derive confidence intervals but the theoretical basis of this approach has not been fully established and it is computationally demanding. A recent addition to the software GenSel that implements genomic selection models [42] has been the use of samples from the MCMC chain to derive posterior distributions of parameters of interest and to use these to identify 1 Mb windows on the genome which contributed more variance than expected under a pure polygenic model in, e.g. 90% of samples of the converged MCMC chain. Initial results from the application of this method to layer chicken data are presented later. The power and impact of alternative window sizes and thresholds have, however, not been evaluated for this method. 

## WHAT HAVE WE LEARNED?

Early QTL mapping studies in livestock, utilizing breed crosses or within-family analyses, identified many QTL regions that were estimated to explain a substantial proportion of phenotypic variance for the trait, depending on the power of the study, with estimated effects greater than 0.2 to 0.3 phenotypic SD, and some with large effects (> 0.5 phenotypic SD) [42]. However, only a limited proportion of these have been validated in independent studies and even fewer have resulted in genetic tests that have been implemented in breeding programs. Until recently, the majority of genetic tests that were used in industry resulted from candidate gene studies and in some cases from genetic tests where the causative mutation was identified [12].

Several studies have attempted a meta-analysis of QTL mapping results to study the genetic architecture of quantitative traits in livestock, accounting for the inherent overestimation of significant effects [44] and reporting bias, i.e. the inability of studies of limited size to identify QTL with smaller effects and the reporting of only significant effects [43,45]. Distributions of QTL effects were generally found to be leptocurtic, with many QTL of small and a few QTL of large (>0.2 or 0.3 phenotypic standard deviations) effect. Results from these studies are, however, sensitive to the distributional assumptions that are made to account for the absence of non-significant QTL effects in the analyses. Weller *et al*. [46] estimated the distribution of effects using estimates at 73 markers across the genome in each of 11 half-sib families without screening for significance. They found that distributions differed substantially between traits, with some traits (female fertility) having few small effects.

Data from high-density SNP genotyping platforms offer new opportunities to study the genetic architecture of quantitative traits, as described previously. One source of information is the comparison of the predictive ability of alternate models used for whole-genome selection. Most simulation studies have found that Bayesian variable selection models that assume a large proportion of SNP to have non-zero effect provide better predictions than models that assume genetic variance is distributed equally across all SNP (GBLUP) [14,47,48]. In these studies, the trait is typically simulated based on at most several hundred QTL with heterogeneous effects. In contrast, most applications of genomic selection models to real data have found that the GBLUP models have very similar predictive ability than the Bayesian variable selection models, except for traits for which there are known genes of large effect, such as fat% in dairy cattle [15,16]. This could be interpreted as evidence that the traits are controlled by a very large number of genes of small effect. Evaluation of these models in real data is, however, typically based on their ability to predict the phenotype of progeny of individuals that are included in the training data. It is well known that such predictions rely heavily on capturing both within and between family relationships [47,48]. In contrast to the historic LD that was the initial premise of genomic selection [14], capturing such effects does not require close linkage between markers and QTL but they can be captured by SNP that are some distance from the QTL and even by SNP across the genome.

More direct information on the genetic architecture of quantitative traits from high-density SNP data comes from analysis of the distribution of the genetic variance contributed by genomic regions. Hayes *et al*. [37] used 50k SNP data from dairy cattle to estimate this distribution for fat%, the proportion of black coat color, and overall type, using windows of 50 SNP. They found that, for all three traits, many segments (up to 96% for % black) explained less than 0.1% of genetic variance, which is the variance expected under a model with equal distribution across the genome. But jointly, these segments explained half of the variance for overall type and proportion black. For all three traits, there were tens of segments that explained 0.1–4.7% of the genetic variance and for the proportion of black and fat% a few segments that explained as much as 37.5% of genetic variance. Several of these coincided with the location of known major genes for these traits. They concluded that, although there are some regions with larger effects, they jointly explain less than 25 to 50% of the genetic variance, depending on the trait. The remaining variance is present in regions spread across the genome.

In a recent analysis to investigate the genetic architecture of traits in layer chickens, we used phenotypic records for egg weight, egg production, and puncture score, a measure of shell strength, on 1,563 chickens from a commercial layer line that were genotyped for 24,430 segregating SNP across the genome. Bayesian variable selection model Bayes-C-π [19], as implemented in the GenSel software [42], was used. In this model the proportion of SNP with zero effect (π) is estimated and the effects of SNP with non-zero effects are assumed to have equal variance a priori. The genome was divided into approximately 1000 windows of 1 Mb and the posterior genetic variance of each window was estimated from samples of the MCMC chain. Results are summarized in Table **1**. Estimates of heritability derived from the marker data were lower than heritabilities estimated using pedigree relationships. Thus, some portion of the genetic variance, up to 1/3 for puncture score, was not accounted for by the SNP. Estimates of π indicated that a large proportion of SNPs had zero effects, over 95% for puncture score and 99% for egg weight and egg production. For egg weight and egg production, less than 5 and 10% of the genome, respectively, was needed to capture over 50% of the marker-based variance, with the rest located in windows with small effects across the genome. For puncture score, the largest effects were smaller and over 32% of the genome was needed to capture over 50% of variance. These results generally agree with those of Hayes *et al*. [37], in that 1) genetic architecture differs between traits, 2) depending on the trait, genes with large effects are present, but 3) over 50% of the genetic variance resides in a large number of genomic regions spread across the genome with effect sizes consistent with a highly polygenic model of inheritance, approaching the infinitesimal model of quantitative genetics. 

## THE FUTURE 

The use of high-density SNP genotypes in whole-genome selection does not require detailed knowledge of the genetic architecture of traits, as methods that assume all SNP contribute equal variance have similar accuracy as the Bayesian variable selection models that use different priors. This is expected to change as SNP density increases, even to the point of having full sequence on large numbers of individuals. This was demonstrated in simulation studies by Meuwissen and Goddard [49], in which they showed that the advantage of predictions obtained from the Bayesian variable selection model Bayes-B increased relative to those from GBLUP with an increase in SNP density, as Bayes-B was able to identify SNP that are in tight LD with the QTL and assign other SNP zero effects. When the causative mutations were included in the available genotypes, as would be expected with availability of full sequence, accuracies however only increased slightly compared to a SNP panel with very high density but no causative mutations. This also implies that, with sufficient data, methods such as Bayes-B will be able to fine map the causative mutations. This can lead to additional advances in management and treatment based on knowledge of genetic mechanisms that underlie important traits.

Current models for genomic selection and GWAS primarily fit additive models but Bayesian variable selection models that fit dominance [50] and even epistatic effects [51] are available or possible. While computationally more demanding, the potential advantage of these additions for predicting breeding values may be limited if, as argued by Hill *et al*. [52], most genetic variance for complex traits can be captured by additive genetic variance. Nevertheless, the use of these models may provide more insight into the genetic architecture of quantitative traits. Also, if genotyping data spans many generations and multiple populations, models that include dominance and epistatic effects may become more important to accurately model the diverse genetic backgrounds that may be represented. Accumulating the required large data sets with high-density SNP data, up to sequence, at a reasonable cost requires intensive and clever use of genotype imputation [53,54], such that most individuals can be genotyped using more cost-effective low-density SNP platforms.

## CONCLUSIONS

Recent developments in molecular and genotyping technology, combined with advances in statistical methodology for the use of this data in prediction of breeding values, has led to development and successful implementation of whole-genome selection methods in dairy cattle. Implementation in other livestock species is underway but is limited by the large training data sets with genotyped and phenotyped animals that are needed. Whole-genome selection models accommodate the highly polygenic genetic architecture of most traits of interest in livestock. Statistical methods used for prediction in whole-genome selection can also be used to identify genomic regions associated with traits and to investigate the genetic architecture of quantitative traits. Results to date show that most traits of interest are indeed highly polygenic.

## Figures and Tables

**Table 1.** Estimates of Genetic Architecture Obtained from Genome-Wide Association Analysis of High-Density SNP Data for Three Traits in a Layer Chicken Line Using Bayesian Variable Selection Model Bayes-C-π

	Egg weight	Egg production	Puncture score
Pedigree-based heritability	0.74	0.39	0.29
Marker-based heritability	0.54	0.32	0.19
% SNP with zero effect (p)	99.2	99.0	95.6
% genome to capture 50% of marker-based variance	4.2	9.9	32.5
% variance of largest window	18.8	3.5	0.8
